# The Association Between Cadmium Exposure and Endometrial Cancer Risk: Evidence from a Comprehensive Updated Meta-Analysis

**DOI:** 10.3390/jcm15041479

**Published:** 2026-02-13

**Authors:** Shiyu Zheng, Xianwei Guo, Xiaoyan Ying

**Affiliations:** 1 Department of Obstetrics and Gynecology, Second Affiliated Hospital of Nanjing Medical University, Nanjing 210003, China; zhengshiyu@stu.njmu.edu.cn; 2 Second Clinical Medical College, Nanjing Medical University, Nanjing 210011, China; 3 Clinical Medical College, Soochow University, Suzhou 215006, China; gxw1997@suda.edu.cn

**Keywords:** cadmium, endometrial cancer, systematic review, meta-analysis

## Abstract

**Background**: The carcinogenic potential of cadmium has been suggested, but its association with endometrial cancer risk remains uncertain. This meta-analysis aimed to evaluate whether cadmium exposure is associated with the risk of endometrial cancer. **Methods**: A thorough search of seven databases was conducted to identify observational studies published up to September 2025. The Newcastle-Ottawa Scale (NOS) and the Agency for Healthcare Research and Quality (AHRQ) tool were utilized to evaluate the quality of observational studies. The I^2^ statistic was calculated to assess heterogeneity among studies. Pooled odds ratios (ORs) and corresponding 95% confidence intervals (CIs) were estimated using a random-effects model. Furthermore, sensitivity analysis, subgroup analysis, and an assessment of publication bias were performed. **Results**: Eight studies involving 196,456 participants were included. Study quality assessment indicated that all included studies were of moderate or high quality. Overall, cadmium exposure was associated with an increased risk of endometrial cancer (OR = 1.27, 95% CI: 1.07–1.50, I^2^ = 64.1%). Stronger associations were observed in case–control studies, European populations, and studies using blood or urinary cadmium biomarkers. The association remained significant in high-quality and adjusted analyses. **Conclusions**: The findings of this meta-analysis suggest a possible association between cadmium exposure and endometrial cancer risk. However, given the observational nature of the included studies, causality cannot be established. Further large-scale, well-designed prospective studies with standardized exposure assessment are needed to clarify this relationship.

## 1. Background

Endometrial cancer represents one of the most prevalent gynecological malignancies worldwide [1]. According to the 2022 GLOBOCAN estimates, it ranks as the 15th most frequently diagnosed cancer, with 420,242 new cases, and it is the 19th leading cause of cancer-related mortality, resulting in 97,704 deaths [2]. Its incidence has been rising steadily over the past several decades, reflecting the interplay of demographic shifts, lifestyle changes, and metabolic disorders [3]. This rising trend not only underscores a growing public health challenge but also highlights the need to identify modifiable risk factors beyond traditional determinants such as obesity, unopposed estrogen exposure, diabetes, and metabolic dysregulation [4,5,6]. Increasingly, environmental exposures have emerged as potentially significant contributors to endometrial carcinogenesis, suggesting that the etiological landscape of this malignancy is more complex than previously recognized [7,8,9].

Among the diverse array of environmental toxicants, cadmium is a naturally occurring heavy metal and a well-established environmental endocrine disruptor, and it has garnered increasing attention for its potential role in reproductive and carcinogenic outcomes [10,11]. Cadmium is widely distributed in the environment, primarily originating from industrial emissions, cigarette smoke, and contaminated food and water [12]. Increasing evidence has linked cadmium to several hormone-dependent malignancies, including breast, prostate, and endometrial cancers, suggesting its broad oncogenic potential [13,14,15]. Mechanistic studies indicate that cadmium exerts its carcinogenic effects via multiple pathways, including induction of oxidative stress, interference with DNA damage response and repair, and estrogen-mimicking activity, thereby promoting tumor initiation and progression [16,17,18]. Despite these mechanistic insights, several epidemiological studies have investigated the association between cadmium exposure and endometrial cancer risk, yet their findings have been inconsistent. For instance, Jiang et al. [19] reported that elevated urinary cadmium levels were associated with an increased risk of endometrial cancer in a population-based study, whereas Eriksen et al. found no significant association among Danish postmenopausal women [20]. Similarly, a previous meta-analysis by Chitakwa et al. did not support a positive association between cadmium exposure and endometrial cancer risk, with the reported pooled estimate failing to reach statistical significance (95% CI: 0.92–1.41) [21]. Since the publication of that meta-analysis, additional studies have been conducted with contrasting results, further highlighting the uncertainty in the current evidence. Given the rising burden of endometrial cancer and the widespread environmental exposure to cadmium, clarifying this association is of significant public health importance [22,23]. Therefore, we performed an updated meta-analysis to comprehensively evaluate the association between cadmium exposure and endometrial cancer risk, aiming to provide more robust and up-to-date evidence.

## 2. Methods

This meta-analysis was conducted in accordance with the Preferred Reporting Items for Systematic Reviews and Meta-Analyses (PRISMA) guidelines and was prospectively registered in PROSPERO (CRD420251154299) [24]. The PRISMA 2020 checklist is provided in Supplementary Material S1. In addition, the completed Meta-analysis Of Observational Studies in Epidemiology (MOOSE) checklist is available in the Supplementary Material S2 [25].

### 2.1. Search Strategy

A thorough search for publications regarding population studies on cadmium exposure and endometrial cancer was performed across four English-language databases (PubMed, Web of Science, Embase, and Cochrane Library) and three Chinese scholarly databases (China National Knowledge Infrastructure, Wanfang Data, VIP Database). The search spanned from the inception of each database to 22 September 2025. No language restrictions were applied. The search terms utilized were (cadmium OR “cadmium exposure”) AND (endometrial OR uterine) AND (cancer OR carcinoma OR neoplasm OR tumor OR malignancy). Furthermore, the reference lists of relevant reviews and articles were scrutinized to identify additional pertinent studies.

### 2.2. Inclusion and Exclusion Criteria

Original studies were included based on the following criteria: (1) study designs comprising case–control studies, randomized controlled trials, cross-sectional studies or cohort studies; (2) studies assessing any exposure to cadmium; (3) endometrial cancer risk as the primary outcome of interest; and (4) studies reporting measures of relative risk (RR), hazard ratio (HR), odds ratio (OR), or incidence rate ratio (IRR) along with their respective 95% confidence intervals (CIs). Studies lacking direct statistical estimates were included if they provided datasets that allowed calculation of effect sizes. The exclusion criteria were: (1) non-original research, including reviews, meta-analyses, editorials, letters, case reports, and conference abstracts; (2) experimental studies (in vivo or in vitro); and (3) duplicate publications or studies based on overlapping populations. Two reviewers (S.Z. and X.G.) independently screened all titles, abstracts, and full-text articles, and disagreements were resolved through discussion with a third reviewer (X.Y.).

### 2.3. Data Extraction and Quality Assessment

Two authors (S.Z. and X.G.) independently extracted relevant data and assessed the quality of the studies. Any discrepancies were resolved through consultation with a third author (X.Y.). A standardized data extraction form was utilized to collect information such as the first author, publication year, country, study design, sample size, age of participants, exposure type, study period, estimates with 95% CIs, adjusted confounders, and study quality. Notably, Cadmium exposure was classified as dietary, blood, or urinary based on the original studies. Dietary cadmium exposure was typically estimated using food frequency questionnaires combined with food cadmium content databases and reflects long-term intake from dietary sources. Blood cadmium levels were used as indicators of recent or ongoing exposure from both dietary and environmental sources, whereas urinary cadmium levels were considered biomarkers of cumulative exposure and body burden over time. These exposure categories were extracted as reported and used for subgroup analyses.

The quality of the included case–control and cohort studies was evaluated using the Newcastle-Ottawa Scale (NOS). The NOS comprises eight items categorized into three domains, namely selection, comparability, and exposure, with a maximum possible score of 9. Scores of 0 to 3 points were classified as low quality, 4 to 6 as medium quality, and 7 to 9 as high quality [26]. Moreover, the Agency for Healthcare Research and Quality (AHRQ) tool was used to evaluate the quality of cross-sectional studies, and this instrument objectively assesses study quality across 11 categories [27].

In addition, the certainty of evidence for the primary outcome was assessed using the Grading of Recommendations Assessment, Development and Evaluation (GRADE) approach with GRADEpro GDT software (Version 4, web-based). The quality of evidence was rated as high, moderate, low, or very low based on study limitations, inconsistency, indirectness, imprecision, and publication bias [28].

### 2.4. Statistical Analysis

We assessed the association between cadmium exposure and endometrial cancer by pooling adjusted effect estimates with 95% CIs. Considering the relatively low absolute risk of endometrial cancer, RRs, HRs, and IRRs were regarded as approximately equivalent to ORs and analyzed accordingly [29,30,31,32]. Statistical heterogeneity among studies was assessed using the I^2^ statistic. Pooled ORs and their corresponding 95% confidence intervals (CIs) were estimated using a random-effects model with the DerSimonian–Laird method [33]. Subgroup analyses were performed based on (1) study design (cohort study vs. case–control study vs. cross-sectional study), (2) country location (United States vs. Europe vs. Asia), (3) exposure (dietary cadmium vs. urinary cadmium vs. blood cadmium), (4) study quality (high vs. moderate), and (5) any adjusted data (adjusted vs. unadjusted). Sensitivity analysis was conducted through the leave-one-out approach, omitting one study at a time [34]. Publication bias was assessed graphically using funnel plots to check for asymmetry, supported by Egger’s test and Begg’s test [35,36]. Statistical analyses were carried out using Stata 14 software, and a *p*-value of <0.05 was considered statistically significant.

## 3. Results

### 3.1. Study Characteristics

**Figure 1:**
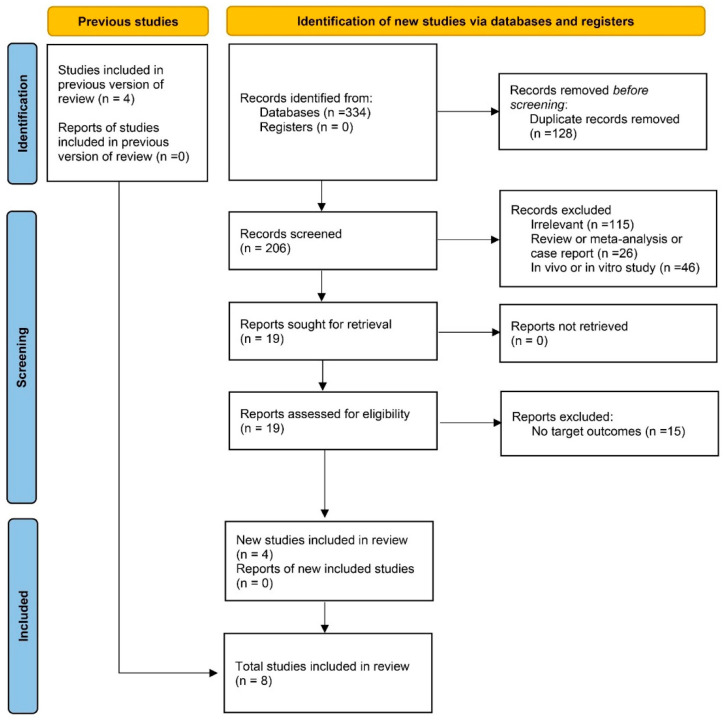
PRISMA flow diagram of included studies.

A total of 334 articles were initially retrieved through our literature search. After duplicate removal, 206 titles and abstracts were screened, and 19 full-text articles were assessed in detail (Figure 1). Based on the predefined eligibility criteria, 15 studies were excluded (Supplementary Material S3). Consequently, four newly eligible studies [19,37,38,39] were included in the current quantitative synthesis, which were combined with the four studies identified in the previous meta-analysis [20,40,41,42]. A total of eight studies [19,20,37,38,39,40,41,42] comprising 196,456 participants, published between 2008 and 2025, were obtained, which included four cohort studies, three case–control studies, and one cross-sectional study. Among the eligible studies, three were conducted in the United States, four were conducted in Europe, and one was from Asia. Seven of them were adjusted for confounders. All articles can achieve high or moderate quality (Supplementary Table S1). All studies reported estimates with 95% CIs as the primary metric for combining the effect size. The summarized characteristics of these studies are presented in Table 1.

### 3.2. Overall Meta-Analysis

**Figure 2:**
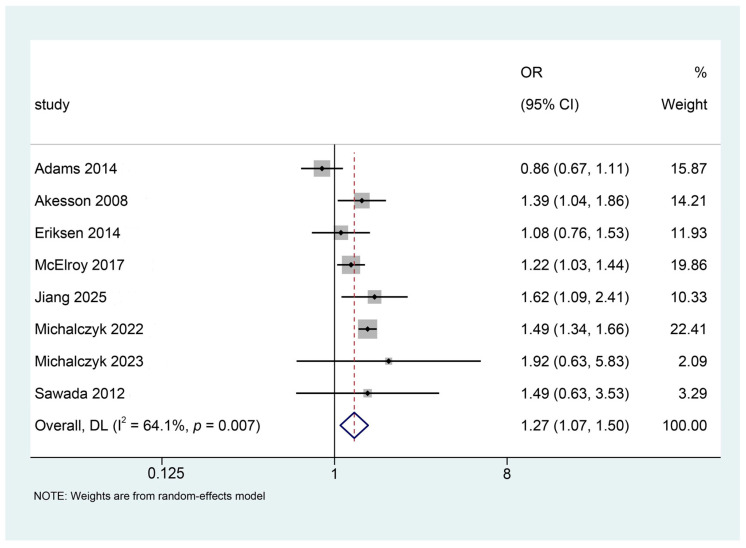
Forest plot of the association between cadmium exposure and endometrial cancer risk. Squares represent individual study estimates (size proportional to weight), horizontal lines indicate 95% confidence intervals, the solid vertical line represents the null value (OR = 1), and the diamond represents the pooled estimate from the random-effects model. References [19,20,37,38,39,40,41,42] are cited in figure.

The eight studies [19,20,37,38,39,40,41,42] included in this analysis examined the association between cadmium exposure and the risk of endometrial cancer. Significant heterogeneity was observed across the studies (I^2^ = 64.1%, P_heterogeneity_ = 0.007). Using a random-effects model, higher cadmium exposure was associated with an increased risk of endometrial cancer (OR = 1.27, 95% CI: 1.07–1.50; *p* = 0.006), as shown in Figure 2. Moreover, according to the GRADE assessment, the certainty of evidence for this association was assessed, and detailed results are presented in the Summary of Findings table (Supplementary Table S2).

### 3.3. Subgroup Analyses

We performed subgroup analyses stratified by study design, country location, type of exposure assessment, study quality, and adjustment status (Table 2). Notably, the association between cadmium exposure and endometrial cancer risk differed markedly by exposure assessment type. When stratified by exposure type, blood cadmium levels were most strongly associated with increased endometrial cancer risk (OR = 1.49, 95% CI: 1.34–1.67, *p* < 0.001), followed by urinary cadmium (OR = 1.32, 95% CI: 1.03–1.70, *p* = 0.029). In contrast, dietary cadmium exposure was not significantly associated with endometrial cancer risk (OR = 1.11, 95% CI: 0.85–1.45, *p* = 0.447). When stratified by study design, case–control studies indicated a significant association between cadmium exposure and endometrial cancer risk (OR = 1.38, 95% CI: 1.16–1.64, *p* < 0.001), while the single cross-sectional study also showed a positive association (OR = 1.62, 95% CI: 1.09–2.41, *p* = 0.018). In contrast, cohort studies did not demonstrate a significant relationship (OR = 1.11, 95% CI: 0.85–1.45, *p* = 0.447). Regarding geographical location, studies conducted in Europe suggested a significantly increased risk (OR = 1.43, 95% CI: 1.27–1.61, *p* < 0.001), while those from the United States did not reach statistical significance (OR = 1.16, 95% CI: 0.86–1.58, *p* = 0.337). The single study from Asia also reported a higher risk (OR = 1.49, 95% CI: 0.63–3.53, *p* = 0.364). In terms of study quality, both high-quality (OR = 1.22, 95% CI: 1.01–1.47, *p* = 0.04) and moderate-quality studies (OR = 1.65, 95% CI: 1.13–2.40, *p* = 0.009) demonstrated significant associations. Finally, when considering adjusted data, studies reporting adjusted estimates suggested a significant association (OR = 1.26, 95% CI: 1.06–1.49, *p* = 0.01), whereas the single study reporting unadjusted findings did not (OR = 1.92, 95% CI: 0.63–5.83, *p* = 0.249).

### 3.4. Sensitivity Analyses and Publication Bias

**Figure 3:**
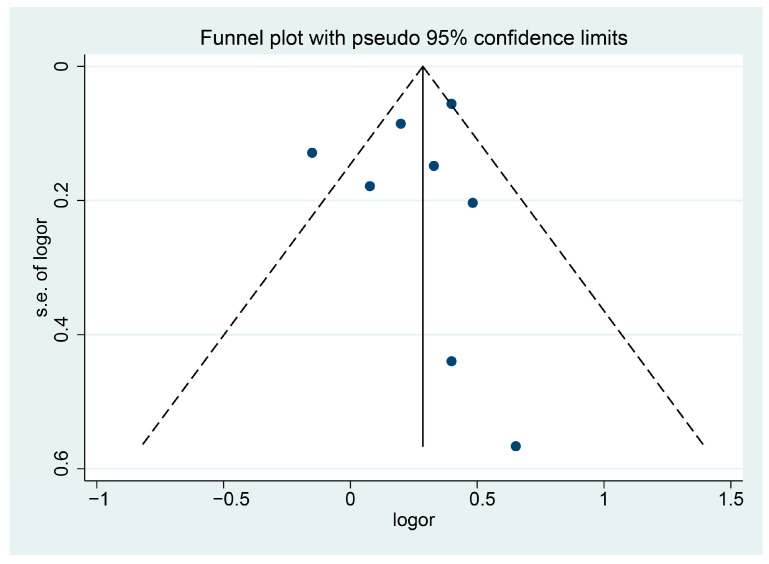
Funnel plot of the association between cadmium exposure and endometrial cancer risk.

Sensitivity analysis revealed that our result was stable. When the included studies were excluded on a one-by-one basis, the ORs were still all greater than 1, and the *p*-value was less than 0.05. The funnel plot (Figure 3) appeared symmetrical, indicating no significant publication bias. Both Begg’s test (Z = 0.12; *p* = 0.902) and Egger’s test (t = −0.50; *p* = 0.638) indicate that there was no evidence of publication bias.

## 4. Discussion

### 4.1. Principal Findings and Subgroup Analyses

In the present study, we performed an updated meta-analysis to comprehensively evaluate the association between cadmium exposure and endometrial cancer risk. Our findings indicate that elevated cadmium exposure may be associated with an increased risk of endometrial cancer. This contrasts with the conclusions of the previous meta-analysis by Chitakwa et al., which reported no statistically significant association [21]. The discrepancy is likely attributable to the inclusion of more recent studies in our analysis, resulting in a larger overall sample size and enhanced statistical power, thereby providing a more updated summary of the available evidence [43]. However, based on the GRADE assessment, the overall certainty of evidence was rated as very low, reflecting the predominance of observational studies and substantial heterogeneity, and the findings should be interpreted cautiously. A key point when interpreting these findings is the variability among the included studies. The strength and direction of the association differed across study designs, exposure measures, and population characteristics, indicating that the pooled odds ratio represents an overall summary signal across heterogeneous studies rather than a uniform effect applicable to all populations and settings. This variation indicates that cadmium may not influence endometrial cancer risk in the same way across different groups, and that factors such as methodological differences, exposure sources, and regional environments may contribute to the observed results.

The subgroup analyses indicated that the observed positive association between cadmium exposure and endometrial cancer was primarily driven by case–control and cross-sectional studies, whereas cohort studies did not demonstrate a significant effect. Although this discrepancy may partly reflect differences in study design and methodological limitations, the overall pattern suggests a possible association between cadmium exposure and endometrial cancer. Nevertheless, additional large-scale prospective cohort studies are needed to further substantiate these findings and strengthen the causal inference. Geographic variation was also evident: studies conducted in European countries consistently reported significant associations, while those from the United States did not. Such heterogeneity could be explained by differences in dietary habits, cadmium contamination levels in food and the environment, and genetic susceptibility across populations [44,45,46]. Regarding exposure type, biomarker-based assessments, particularly blood and urinary cadmium, demonstrated stronger associations with endometrial cancer risk than dietary intake. The limitations of dietary cadmium exposure assessment should be explicitly acknowledged, as such assessments rely largely on food frequency questionnaires and cadmium content databases, both of which are prone to substantial exposure misclassification [47,48]. These methods may inadequately capture individual variability in cadmium intake as well as long-term internal cadmium burden, which may contribute to attenuation of observed associations. In contrast, blood and urinary cadmium reflect cumulative exposure from multiple sources and provide a more accurate measure of biologically relevant internal dose [44]. This distinction may explain why biomarker-based measures showed stronger and more consistent associations and underscores the importance of using objective exposure assessments in future studies. Both high- and moderate-quality studies yielded significant associations, indicating that the observed effect is unlikely to be entirely attributable to study quality.

### 4.2. Potential Biological Mechanisms

**Figure 4:**
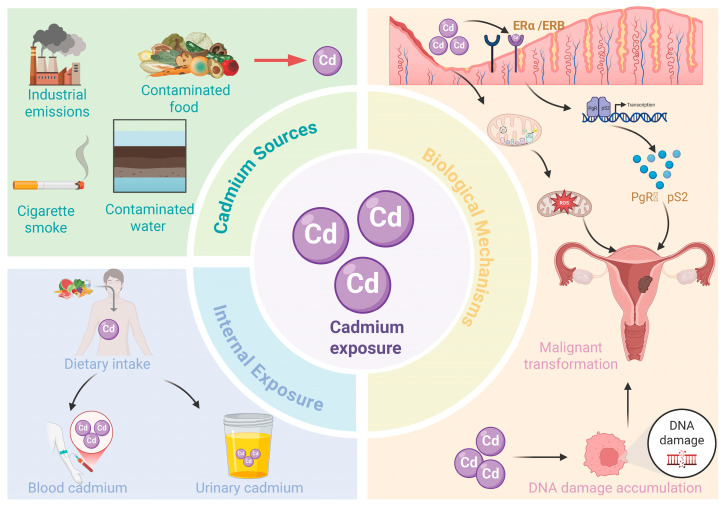
Simple schematic of the pathway from cadmium sources to exposure, biological effects, and endometrial cancer.

Previous studies investigating the mechanisms underlying cadmium-induced endometrial cancer have proposed multiple potential pathways to explain these findings. First, Cadmium is recognized as a potential metalloestrogen, capable of mimicking estrogenic activity in human tissues. The endometrium, which expresses high levels of estrogen receptors ERα and ERβ, is a potential target for cadmium accumulation and interaction [49]. Studies have demonstrated that cadmium can activate ERα by binding to its hormone-binding domain, leading to transcriptional activation of estrogen-responsive genes such as PgR and pS2 [50,51]. These genes are crucial regulators of cell proliferation and differentiation, and their dysregulated expression may result in uncontrolled endometrial cell growth. Therefore, it can be speculated that cadmium exposure may contribute to neoplastic transformation in the endometrium and increase the risk of endometrial cancer. Second, cadmium-induced oxidative stress plays a critical role in driving carcinogenesis. As a heavy metal ion, cadmium accelerates the production of reactive oxygen species (ROS) in endometrial cells by disrupting mitochondrial electron transport chains and inhibiting antioxidant enzymes [52]. Excessive ROS accumulation not only causes oxidative damage to lipids, proteins, and nucleic acids but also exacerbates cellular damage and hinders apoptotic clearance of abnormal cells [53]. Collectively, these interrelated mechanisms underscore the multifactorial role of cadmium in promoting endometrial carcinogenesis, although further studies are warranted to fully elucidate the underlying pathways (Figure 4).

### 4.3. Strengths

This study has several notable strengths. First, in comparison with previous meta-analyses, our work incorporated a greater number of eligible studies, which not only enhanced the statistical power but also allowed for a more refined and comprehensive subgroup analysis across different study designs, populations, and exposure assessments. Second, the robustness of our findings was further supported by sensitivity analyses, which consistently confirmed the relative stability of the pooled estimates. Finally, the majority of effect estimates were based on multivariable-adjusted models, thereby reducing the influence of potential confounding factors and strengthening both the accuracy and credibility of the results [54]. Collectively, these strengths highlight the methodological rigor of our meta-analysis and provide a solid foundation for interpreting the associations observed between cadmium exposure and endometrial cancer risk. Confounding is an important consideration. Although most studies reported adjusted estimates, the covariates differed, with some adjusting for key factors such as smoking, BMI, reproductive history, hormone use, and socioeconomic status, while others included only a subset. This inconsistency may contribute to heterogeneity, and residual confounding cannot be ruled out. Therefore, the pooled estimates should be interpreted cautiously, as unmeasured or incompletely controlled confounders may still influence the results.

### 4.4. Limitations

Despite these strengths, several limitations should be acknowledged. First, the number of eligible studies was relatively limited, which constrained the robustness of the overall findings despite combining different exposure indicators, including dietary cadmium intake, blood cadmium levels, and urinary cadmium levels. To partially address this, we performed subgroup analyses to explore sources of heterogeneity. Second, all included studies were observational, limiting our ability to establish causal relationships or fully elucidate the underlying mechanisms. In particular, evidence from cohort studies remains insufficient, and the small number of studies precluded meaningful meta-regression analyses; therefore, potential sources of heterogeneity were discussed narratively rather than through statistically underpowered methods. Third, the limited data prevented more detailed subgroup analyses based on factors such as menopausal status (premenopausal vs. postmenopausal), which may have further clarified heterogeneity. Fourth, as fewer than 10 studies were available, the results of publication bias assessments using Egger’s and Begg’s tests may have limited statistical power and should therefore be interpreted as exploratory. Fifth, the inclusion of different study designs and effect measures introduces methodological variability. Cross-sectional studies estimate prevalence rather than incidence and are more susceptible to survival bias and reverse causation, while harmonizing ORs, RRs, HRs, and IRRs under the rare-disease assumption may be less valid in older or high-risk populations [55]. Moreover, ORs may overestimate the magnitude of associations, particularly when the outcome is not rare, which could have introduced additional bias into the pooled estimates [29]. These differences complicate causal inference and contribute to heterogeneity; therefore, the pooled estimate should be interpreted as a broad summary rather than a precise effect applicable to all settings. Finally, the lack of a significant association in cohort studies restricts causal interpretation and suggests that the pooled estimate should be interpreted with caution, as the observed association was mainly derived from case–control and cross-sectional studies.

## 5. Conclusions

In summary, this updated meta-analysis indicates that cadmium exposure may be associated with an increased risk of endometrial cancer. These findings may provide useful insight for future research on environmental risk factors but should be regarded as hypothesis-generating rather than conclusive, as the available evidence is derived exclusively from observational studies and does not allow causal inference. Further large-scale, well-designed prospective studies with standardized exposure assessment are required to clarify this association.

## Figures and Tables

**Table 1:** Characteristics of studies included in this meta-analysis.

Study	Study Design	Country	Sample Size	Age (Years)	Exposure	Study Period	Estimate (95% CI)	Quality	Adjustment Factors
Adams 2014 [40]	Cohort study	United State	91,643	50–79	Dietary cadmium	1993–1998	HR: 0.86 (0.67–1.11)	High	Adjusted ^a^
Akesson 2008 [41]	Cohort study	Sweden	30,210	Mean age: 60.4	Dietary cadmium	1987–1990	RR: 1.39 (1.04–1.86)	High	Adjusted ^b^
Eriksen 2014 [20]	Cohort study	Denmark	22,279	50–65	Dietary cadmium	1993–1997	IRR: 1.08 (0.76–1.53)	High	Adjusted ^c^
McElroy 2017 [42]	Case–control study	United State	1510	18–81	Urinary cadmium	2010–2012	OR: 1.22 (1.03–1.44)	High	Adjusted ^d^
Jiang 2025 [19]	Cross-sectional study	United State	2802	Over 20	Urinary cadmium	2003–2018	OR: 1.62 (1.09–2.42)	Moderate	Adjusted ^e^
Michalczyk 2022 [37]	Case–control study	Poland	140	NA	Blood cadmium	NA	OR: 1.49 (1.31–1.63)	High	Adjusted ^f^
Michalczyk 2023 [38]	Case–control study	Poland	110	Median age: 52	Blood cadmium	NA	OR: 1.92 (0.63–5.8)	Moderate	Unadjusted
Sawada 2012 [39]	Cohort study	Japan	47,762	NA	Urinary cadmium	1993–1998	HR: 1.49 (0.63–3.53)	High	Adjusted ^g^

^a^ Adjusted for total energy intake, age, study component, BMI, smoking, alcohol consumption, race, education, physical activity, age at first birth, age at menarche, age at menopause, unopposed estrogen use, and estrogen and progesterone use. ^b^ Adjusted for attained age in years, postsecondary education, parity, age at menarche, age at menopause, leisure time physical inactivity, BMI, use of postmenopausal hormones, smoking status, and total amount of vegetables, whole grains, and potatoes intakes. ^c^ Adjusted for educational level, smoking, number of births, age at first birth, hormone replacement therapy status, hormone replacement therapy use, age at menarche, BMI, height, physical activity, and alcohol intake. ^d^ Adjusted for urinary creatinine, race, marital status, BMI, history of trying to lose weight, smoking, history of endometriosis, history of breast cancer, history of ovarian cancer, history of uterine fibroids, endometrial cancer in first degree relative, oral contraceptive use, unopposed estrogen use, menopause at age, post-menopausal at diagnosis, protein shake consumption, and whole milk consumption. ^e^ Adjusted for urinary creatinine, BMI, smoking, alcohol consumption, menarche age years, menopause age years, pregnant status, hysterectomy, oophorectomy, and oral contraceptives. ^f^ Adjusted for menopause, grading, staging, smoking, BMI, diabetes, Hashimoto, Cu, Zn, Pb, Co, and P. ^g^ Adjusted for age, area, BMI, smoking, frequency of alcohol intake, leisure-time physical activity, intake of meat, soybean, vegetable, fruit, menopausal status, and use of exogenous female hormones. BMI: Body mass index; HR: Hazard ratio; IRR: Incidence rate ratios; NA: Not applicable; OR: Odds ratio; RR: Relative ratio.

**Table 2:** Results of subgroup analyses in the Meta-Analysis.

Analysis	No. of Studies	OR (95% CI)	P_effect_	Heterogeneity (I^2^)	P_heterogeneity_	Model
All studies	8	1.27 (1.07–1.50)	0.006	64.1%	0.007	Random
Study design						
Cohort studies	4	1.11 (0.85–1.45)	0.447	54.2%	0.087	Random
Case–control studies	3	1.38 (1.16–1.64)	<0.001	51.7%	0.126	Random
Cross-sectional study	1	1.62 (1.09–2.41)	0.018	NA	NA	Random
Country location						
United States	3	1.16 (0.86–1.58)	0.337	76.2%	0.015	Random
European countries	4	1.43 (1.27–1.61)	<0.001	8.6%	0.350	Random
Eastern country	1	1.49 (0.63–3.53)	0.364	NA	NA	Random
Exposure						
Dietary cadmium	4	1.11 (0.85–1.45)	0.447	54.2%	0.087	Random
Urinary cadmium	2	1.32 (1.03–1.70)	0.029	39.4%	0.199	Random
Blood cadmium	2	1.49 (1.34–1.67)	<0.001	0	0.656	Random
Study quality						
High	6	1.22 (1.01–1.47)	0.040	72.4%	0.003	Random
Moderate	2	1.65 (1.13–2.40)	0.009	0	0.778	Random
Any adjusted data						
Adjusted findings	7	1.26 (1.06–1.49)	0.010	68.6%	0.004	Random
Unadjusted findings	1	1.92 (0.63–5.83)	0.249	NA	NA	Random

NA: Not applicable.

## Data Availability

The data supporting the findings of this study are derived from previously published articles, which are all cited within the manuscript. No new data were created or analyzed in this study. Data sharing is therefore not applicable.

## Supplementary Materials

The following supporting information can be downloaded at https://www.mdpi.com/article/10.3390/jcm15041479/s1, Supplementary Material S1: PRISMA 2020 checklist [56]; Supplementary Material S2: MOOSE checklist; Supplementary Material S3: Studies excluded after full-text review (*n* = 15). Table S1: Quality assessment; Table S2: GRADE assessment.

## Abbreviations

The following abbreviations are used in this manuscript:

AHRQ	Agency for Healthcare Research and Quality
HR	Hazard ratio
IRR	Incidence rate ratio
MOOSE	Meta-analysis Of Observational Studies in Epidemiology
NOS	Newcastle-Ottawa Scale
OR	Odds ratio
RR	Relative risk
PRISMA	Preferred Reporting Items for Systematic Reviews and Meta-Analyses
PROSPERO	Prospective Register of Systematic Reviews
