# Supplementary material for: The Association Between Cadmium Exposure and Endometrial Cancer Risk: Evidence from a Comprehensive Updated Meta-Analysis
Source: J Clin Med. 2026 Feb 13;15(4):1479. doi: 10.3390/jcm15041479 (PMC12941729; doi:10.3390/jcm15041479)
Supplement: Supplementary file 1 [file jcm-15-01479-s001.zip › Supplementary material S3 List of the studies excluded after full-text review.pdf]

**Supplementary material 3**. Studies excluded after full-text review (n = 15)

| First author (Year) | Title | DOI | Reason |
|---|---|---|---|
| Balasubramaniyan (1994) | Involvement of plasma copper, zinc and cadmium in human carcinoma of uterine cervix | 10.1007/BF02999866 | The study focused on cervical cancer and did not assess endometrial cancer risk. |
| Delaney (2016) | Whole Genome Pathway Analysis Identifies an Association of Cadmium Response Gene Loss with Copy Number Variation in Mutant p53 Bearing Uterine Endometrial Carcinomas | 10.1371/journal.pone.0159114 | The analysis was limited to genomic alterations within cancer cases and did not evaluate disease risk. |
| Guyot (2015) | Determination of Heavy Metal Concentrations in Normal and Pathological Human Endometrial Biopsies and In Vitro Regulation of Gene Expression by Metals in the Ishikawa and Hec-1b Endometrial Cell Line | 10.1371/journal.pone.0142590 | The study compared metal concentrations in tissue samples without assessing cancer risk. |
| Haruna (2025) | The Combined Effects of Urine Zinc, Cadmium, Mercury, Lead, and Copper on Endometrial Cancer Staging | 10.3390/ijerph22020245 | The study focused on cancer staging among cases and did not evaluate endometrial cancer risk. |
| Nasiadek (2005) | Tissue levels of cadmium and trace elements in patients with myoma and uterine cancer | 10.1191/0960327105ht575oa | The study was based on tissue concentration comparisons and did not assess disease risk. |
| Nasiadek (2013) | The effect of cadmium on the coagulation and fibrinolytic system in women with uterine endometrial cancer and myoma | 10.2478/s13382-013-0089-z | The outcomes were coagulation markers in patients rather than cancer risk. |

| | | | |
|---|---|---|---|
| Pochwałowski (2001) | Cadmium concentration in uterine myomas, myometrium and peripheral blood from women living in Lower Silesia | PMID: 11883208 | The study described metal concentrations without evaluating endometrial cancer risk. |
| Tomczyk (2022) | Canonical analysis of concentrations of toxic metals in endometrium of women with gynecological disorders | 10.5603/GP.a2022.0088 | The study analyzed tissue metal patterns across diagnoses and did not estimate cancer risk. |
| Wieder-Huszla (2022) | Evaluation of the Concentration of Selected Elements in Patients with Cancer of the Reproductive Organs with Respect to Treatment Stage-Preliminary Study | 10.3390/nu14122368 | The analysis focused on treatment stage and element levels rather than disease risk. |
| Yaman (2007) | Comparison of trace element concentrations in cancerous and noncancerous human endometrial and ovary tissues | 10.1111/j.1525-1438.2006.00742.x | The study compared tissue concentrations and did not assess endometrial cancer risk. |
| Wadhwa (2015) | Interaction between carcinogenic and anti-carcinogenic trace elements in the scalp hair samples of different types of Pakistani female cancer patients | 10.1016/j.cca.2014.10.007 | The study evaluated trace elements among cancer patients without risk analysis. |
| Yang (2025) | Association between blood heavy metals exposure with uterine fibroids among American women: a cross-sectional analysis from NHANES data | 10.1186/s12905-025-03596-4 | The analysis focused on uterine fibroids rather than endometrial cancer risk. |
| Geller (2025) | A Prospective Ultrasound Study of Whole Blood Metals and Incidence of Uterine Leiomyomata | 10.1289/EHP15218 | The outcome of interest was uterine leiomyomata risk but not endometrial cancer risk. |

| | | | |
|---|---|---|---|
| Razumova (2022) | Cadmium Intake as a Prognostic Factor in Endometrial Cancer | 10.1080/01635581.2021.1883681 | The study focused on prognosis and survival rather than cancer risk. |
| Jackson (2008) | The association between heavy metals, endometriosis and uterine myomas among premenopausal women | 10.1093/humrep/dem394 | The outcomes were endometriosis and uterine myomas rather than endometrial cancer. |
