# Supplementary material for: The Association Between Cadmium Exposure and Endometrial Cancer Risk: Evidence from a Comprehensive Updated Meta-Analysis
Source: J Clin Med. 2026 Feb 13;15(4):1479. doi: 10.3390/jcm15041479 (PMC12941729; doi:10.3390/jcm15041479)
Supplement: Supplementary file 1 [file jcm-15-01479-s001.zip › Supplementary Table S1 Quality assessment.pdf]

Supplementary Table S1 Detailed quality assessment of included studies

Part A Newcastle–Ottawa Scale (NOS) assessment for cohort and case–control studies

Study	Study design	Selection				Comparability	Outcome/Exposure			Score
		Item 1	Item 2	Item 3	Item 4	Item 1	Item 1	Item 2	Item 3	
Adams 2014	Cohort study	1	1	1	0	2	1	1	1	8
Akesson 2008	Cohort study	1	1	1	0	2	1	1	1	8
Eriksen 2014	Cohort study	1	1	1	0	2	1	1	1	8
McElroy 2017	Case-control study	1	1	0	1	2	1	1	0	7
Michalczyk 2022	Case-control study	1	1	0	1	2	1	1	0	7
Michalczyk 2023	Case-control study	1	1	0	1	0	1	1	0	5
Sawada 2012	Cohort study	1	1	1	1	2	1	1	1	8

Part B AHRQ assessment for cross-sectional study

Author	Study Design	Item 1	Item 2	Item 3	Item 4	Item 5	Item 6	Item 7	Item 8	Item 9	Item 10	Item 11
Jiang 2025	Cross-sectional study	Yes	Yes	No	Yes	No	No	No	Yes	Yes	No	Yes
