# Supplementary material for: The Association Between Cadmium Exposure and Endometrial Cancer Risk: Evidence from a Comprehensive Updated Meta-Analysis
Source: J Clin Med. 2026 Feb 13;15(4):1479. doi: 10.3390/jcm15041479 (PMC12941729; doi:10.3390/jcm15041479)
Supplement: Supplementary file 1 [file jcm-15-01479-s001.zip › Supplementary Table S2 GRADE assessment.pdf]

Author(s): Zheng S; Guo X; Ying X
Question: Cadmium exposure compared to no cadmium exposure for Endometrial cancer risk.
Setting: General population
Bibliography:

Certainty assessment							№ of patients		Effect		Certainty	Importance
№ of studies	Study design	Risk of bias	Inconsistency	Indirectness	Imprecision	Other considerations	Cadmium exposure	no cadmium exposure	Relative (95% CI)	Absolute (95% CI)		
**New outcome**												
8	non-randomised studies	not serious	serious[a]	not serious	not serious	none			**OR 1.27** (1.07 to 1.50)	**-- per 1,000** (from -- to --)[b]	⊕□□□ Very low[a]	CRITICAL

CI: confidence interval; **OR:** odds ratio

Explanations

a. Serious inconsistency was noted due to substantial heterogeneity across studies (I^2 = 64%), likely reflecting differences in study design, exposure assessment methods, and study populations.
b. Absolute effects were not calculated because a reliable and uniform baseline risk could not be established across the included observational studies, making the estimation of absolute risk differences inappropriate.
